# The Effect of Cold Application to the Lateral Neck Area on Peripheral Vascular Access Pain: A Randomised Controlled Study

**DOI:** 10.3390/jcm12196273

**Published:** 2023-09-28

**Authors:** Senay Canikli Adıgüzel, Dilan Akyurt, Gökçe Ültan Özgen, Hatice Bahadır Altun, Aleyna Çakır, Mustafa Süren, İsmail Okan

**Affiliations:** 1 Samsun Training and Research Hospital, Samsun University, 55090 Samsun, Turkey; dilanakyurt@gmail.com (D.A.); gokce_ultan@yahoo.co.uk (G.Ü.Ö.); haticebahadirmd@hotmail.com (H.B.A.); aleynackr63@gmail.com (A.Ç.); mustafa.suren@samsun.edu.tr (M.S.); 2 İstanbul Medeniyet University, 34720 Istanbul, Turkey; ismail.okan@medeniyet.edu.tr

**Keywords:** vascular access pain, heart rate, cold application, vagal stimulation

## Abstract

Introduction: Various types of vagus nerve stimulation are employed in the treatment of a range of conditions, including depression, anxiety, epilepsy, headache, tinnitus, atrial fibrillation, schizophrenia, and musculoskeletal pain. The objective of this study was to apply vagal stimulation to the neck area using standardised cold, and then analyse the level of vascular access discomfort experienced by individuals who underwent venous cannulation from the dorsal side of the hand prior to anaesthesia. Materials and Methods: A total of 180 patients, aged 18–75, who were scheduled to undergo elective surgery, were categorised into three distinct groups: the Sham group (Group S), the Control group (Group K), and the Cold group (Group M), with each group consisting of 60 individuals. Bilateral cold application to the lateral side of the neck was performed prior to the commencement of vascular access in Group M patients, followed by the subsequent opening of vascular access. The alterations in heart rate among patients was assessed subsequent to the application of cold and following the establishment of vascular access. The participants were instructed to assess their level of vascular access pain on a numerical pain scale (NRS) ranging from 0 to 10. Results: A statistically significant difference (*p* = 0.035) was seen when comparing the pain ratings of patients during vascular access. The study revealed that the NRS values exhibited a statistically significant decrease in Group M compared to both Group K (*p* = 0.038) and Group S (*p* = 0.048). Group M had a higher prevalence of individuals experiencing mild pain compared to other groups, and the difference was statistically significant (*p* = 0.029). In Group M, the average heart rate following vagal stimulation exhibited a statistically significant decrease compared to the average heart rate observed at the beginning of the study (*p* < 0.05). Upon comparing the original heart rate measurements with the heart rate values following vascular access, it was observed that there was an elevation in heart rate for both Group S and Group K. Conversely, Group M exhibited a decrease in heart rate after vascular access when compared to the initial heart rate values. Conclusions: In the present investigation, it was discovered that the application of cold to the neck region resulted in a drop in heart rate among the patients, which persisted throughout the process of vascular access. Furthermore, the level of pain experienced by these individuals was reduced during vascular access procedures.

## 1. Introduction

The vagus nerve (N. vagus) extends from the brain stem to the proximal two-thirds of the large intestine and is therefore the longest of the cranial nerves [1]. Furthermore, apart from serving as the primary neuron of the autonomic parasympathetic system, it also has anti-inflammatory and analgesic characteristics. The afferent branch of the N. vagus can be found in the region of the ear known as the ‘cymba conchae’, while the cervical branch is densely situated in the neck region [2]. These regions are employed to deliver stimuli for the vagus stimulation approach. The vagus stimulation approach, formerly employed for managing refractory epilepsy, has been found to have pain-reducing effects in musculoskeletal disorders [3]. Similarly, Eastern medicine has, for millennia, harnessed the analgesic properties of auricular acupuncture [4].

The clinical application of vagus stimulation extends beyond the treatment of depression, epilepsy, and vascular access pain in encompassing additional conditions such as headache, tinnitus, atrial fibrillation, schizophrenia, and various pain management approaches. Transcutaneous cervical vagus stimulation (TCVS) is a frequently utilised non-invasive method [5]. The Valsalva manoeuvre is a physiological technique that elicits activation of the vagus nerve. It is believed that the activation of the vagal nerve by the stimulation of the baroreceptor reflex arc leads to the release of a substance similar to substance P, which exhibits antinociceptive properties [6,7,8]. The application of ice to the lateral neck region is believed to elicit stimulation of the vagus nerve, resulting in a reduction in heart rate. The application of cold to the neck region also leads to an antinociceptive effect, which is attributed to the activation of the parasympathetic system by the stimulation of the baroreceptor reflex arc via vagal stimulation [7].

Intravenous (IV) cannulation is a frequently employed procedure conducted by anaesthesiologists both within and beyond the confines of the operating room. Prior to the initiation of any surgery, it is imperative to establish vascular access. The process of venous cannulation is associated with a moderate level of pain, causing discomfort and heightened stress levels among patients [9]. During the venous cannulation procedure, patients’ stress level and pain may increase, especially in repeated interventions. For this reason, ultrasonography support is also used to increase the success rate of cannulation and aid in single-attempt success [10,11]. Numerous methodologies have been employed in attempts to mitigate the discomfort associated with venous cannulation and to alleviate the patient’s pain and divert their attention. These include the administration of local anaesthetic through injection to the intervention site, the application of topical anaesthetic, the use of cold, and the use of a vibrating buzzy device. The administration of local anaesthetic mostly alleviates the physical aspect of pain, whereas the Valsalva manoeuvre, which involves the stimulation of the vagus nerve, mitigates both the somatic and psychological dimensions of pain [8]. The induction of antinociception can be achieved through the activation of the cardiopulmonary baroreceptor reflex arc or sino-aortic baroreceptor reflex arc via the Valsalva manoeuvre. It has been noted that the elevation of intrathoracic pressure during this manoeuvre leads to a reduction in venous return, thereby underscoring the significance of venous cannulation [12,13].

Aim: The aim of this study was to administer vagus stimulation with the application of standardised cold to the neck area, and thereafter assess the level of pain experienced by patients who underwent venous cannulation on the dorsal side of the hand.

## 2. Materials and Methods

This single-centre study was conducted within the operating room of Samsun University Training and Research Hospital, Samsun, Turkey) between 18 June and 30 July 2023 following ethics committee approval (Decision no. 11/19 of SÜKAEK-07.06.2023) and Clinical Trials (NCT05920915) registration. Upon their arrival at the operating theatre on the designated day of surgery, patients who had already undergone preoperative preparation for the surgical procedure and administration of anaesthesia were briefed about the aims of the study and informed consent was obtained.

Inclusion Criteria: The study included individuals aged between 18 and 70 years who underwent elective surgery and were classified by the American Society of Anesthesiologists (ASA) as class I-III.

Exclusion Criteria: The study excluded individuals who exhibited the following characteristics: a scar located on the dorsal aspect of the hand, a diagnosis of psoriasis, peripheral vascular disease, chronic analgesic usage, opioid usage, steroid usage, gabapentinoid usage, a history of substance and alcohol use, peripheral neuropathy, ongoing oncological treatment, individuals scheduled for oncological surgery, pregnant women, patients using anti-arrhythmic medication, and patients with limited cooperation.

**Figure 1:**
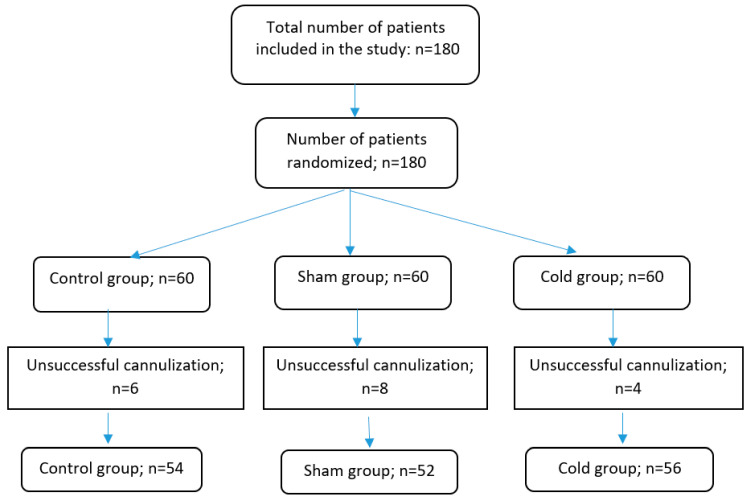
Flow chart of the study. n = number.

Randomisation and intervention: Individuals were transported to the preoperative waiting area where they underwent routine monitorisation. The sample size for each group was determined to be 53 patients, with an additional 7 individuals included for potential losses, resulting in a total sample size of 60 individuals for each group. Patients were randomly assigned to three groups: a Control group (Group K), a Cold group (Group M), and a Sham group (Group S). Randomisation was conducted using the Sequentially Numbered, Opaque, Sealed Envelope (SNOSE) technique [14]. Patients whose venous cannulation from the dorsum of the left hand could not be successfully completed in a single attempt were excluded from the study (Figure 1). 

Group K (Control group): A waiting period of 30 s was observed prior to opening the vascular access, without any intervention. 

Group M (Cold group): Immediately prior to the initiation of vascular access, a marble stone measuring 4 × 5 cm was applied for a duration of 30 s to the carotid artery, specifically positioned 2–3 cm above the collarbone on the sternocleidomastoid muscle (SCM). This marble-derived stone was retrieved from the vegetable shelf of a refrigerator. The stone was subjected to a cooling process at a temperature of −8 °C for a duration of 10 min. Subsequently, the temperature reduced to 11 °C. The temperature of the sample in contact with the palm increases to 12 °C during a span of 1 min and rises to 18 °C over a period of 5 min while placed on a table at room temperature. The marble stone was taken out of the refrigerator at the time of application for each patient and used without waiting.

**Figure 2:**
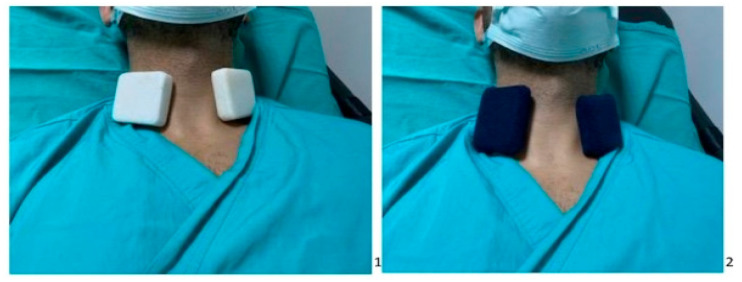
Picture on the left (**1**) is the application of cold marble to the neck area, picture on the right (**2**) is application of non-cold marble to the neck area.

Group S (Sham group): Prior to initiating vascular access, the bilateral neck region of the patients was prepared by applying a 4 × 5 cm marble stone contained within a polar sheath for a duration of 30 s (Figure 2).

Prior to establishing vascular access, the heart rate (HR), respiratory rate (RR), non-invasive blood pressure (NIBP), and oxygen saturation (SPO2) were measured in all patients. The same parameters were then re-measured following 30 s of contact with a marble stone on the neck area in Group M and Group S. In Group K, the measurements were taken after a 30 s waiting period without any intervention. Subsequently, a single insertion of an 18 gauge (green) intravenous catheter was performed by the same healthcare professional on the dorsal aspect of the left hand. The data of heart rate (HR), respiratory rate (RR), non-invasive blood pressure (NIBP), and peripheral capillary oxygen saturation (SpO2) were promptly documented after the placement of the intravenous catheter. The participants were requested to assess their levels of pain on a scale ranging from 0 to 10 using the numerical pain scale (NRS). The records were recorded by the same nurse blinded to the study.

Statistical Analysis: The data were analysed using the SPSS 21 software. GPower 3.1 was utilised to determine the total sample size required for a study with three groups. The effect size was specified as 0.25, the α error was set at 0.05, and the desired power was 0.80. The computed sample size was found to be 159. The study assessed the NRS scores of three groups and investigated the correlation between heart rate (HR), respiratory rate (RR), and non-invasive blood pressure (NIBP), as well as the oxygen saturation (SPO2) readings of the patients before, immediately after, and following the application of a marble. The normality of distribution was assessed using the Kolmogorov–Smirnov and Shapiro–Wilk tests. The examination of data with a normal distribution involved the utilisation of both the analysis of variance (ANOVA) and the paired samples test. The Kruskal–Wallis, Mann–Whitney U, and Wilcoxon Signed Rank tests were used to analyse data that did not follow a normal distribution. The Chi-square test was conducted by constructing cross tables. Descriptive statistics utilised mean ± standard deviation and percentiles. The results were evaluated using a confidence interval of 95% and a significance level of *p* 0.05.

## 3. Results

Data from 162 patients were analysed: 54 in the Control group, 52 in the Sham group, and 56 in the Cold group. The age, gender, and BMI distributions of the patients were comparable between groups (Table 1). No parasympathetic activity-related complications, such as syncope or hypotension, were observed in any of the patients.

The initial values for heart rate, respiratory rate, and non-invasive blood pressure were comparable between groups. After vagal stimulation, the mean heart rate in Group M was significantly lower than at baseline (*p* < 0.05). When the initial heart rate values were compared to the heart rate values after vascular access, there was an increase in the heart rate in Groups S and K, while there was a decrease in the heart rate in Group M (Table 2).

When comparing the pain levels of patients undergoing vascular access, the difference between the groups was statistically significant (*p* = 0.035). Significantly reduced NRS values were observed in Group M compared to Group K (*p* = 0.038) and in Group M compared to Group S (*p* = 0.048). When the NRS value of 1–3 was classified as mild pain and 4–10 as moderate to severe pain, significantly more Group M patients experienced mild pain than Group K patients. Table 3 demonstrates that the number of patients with moderate and severe pain was considerably greater in Group K than in Group M (*p* = 0.029).

When the respiratory rate and non-invasive blood pressure measurements of the patients were evaluated at baseline, prior to vagal stimulation, after vagal stimulation, and after intravenous access, there was no difference between the groups or within the groups.

## 4. Discussion and Conclusions

The aim of this study was to evaluate the level of discomfort experienced by individuals undergoing venous cannulation following vagal stimulation induced by the administration of a cold compress to the neck. Our findings indicate that the individuals in Group M, where cold was applied, exhibited less discomfort during vascular access and a better rate of successful vascular access. A notable reduction in the heart rates of the patients in the cold group was noticed in comparison to their initial levels.

Various Valsalva techniques, namely, balloon insufflation, coughing, and breath-holding, are employed for the purpose of stimulating the vagus nerve [12,13,15]. An alternative method of stimulating the vagus nerve involves the application of cold to the cervical region. Jungman previously demonstrated that the application of cold to the neck region results in a reduction in heart rate [7]. In this study, we found that the heart rate of the patients who underwent cold application to the neck region exhibited a statistically significant decrease compared to their initial values. This decrease was seen both immediately after the application of cold and during vascular access. These findings confirm the occurrence of vagal stimulation.

Patients frequently experience discomfort during vascular access, especially when under the stress of a surgical operation. The venous cannulation process is a crucial component of surgical interventions for patients, and successfully navigating this stage without complications brings significant relief. The NRS values observed during vascular access procedures were found to be lower in the group of patients who had undergone cold therapy compared to the other groups. This difference was deemed to be clinically significant, as seen in Table 3. In a study utilising balloon insufflation for vagus stimulation, a substantial proportion of patients reported the absence of any pain, and a much lower number of patients had severe discomfort [12]. Another study demonstrated that patients who underwent plateletpheresis experienced reduced levels of needle insertion pain and anxiety when utilising the Valsalva manoeuvre [15]. In another study, the effect of the Valsalva manoeuvre on the sensation of pain during the insertion of a catheter during spinal anaesthesia was investigated. No patients in the Valsalva group exhibited severe pain, and the proportion of patients who did not report pain was much greater compared to the other groups. In our study, one patient subjected to cold treatment did not report any pain sensation, whereas a total of 21 individuals had mild pain. In their study, Höbek et al. [16] employed the Valsalva manoeuvre as a means to mitigate the vascular access pain experienced by pregnant women. The researchers noted a decrease in pain levels among the participants. The analgesic effect in various clinical scenarios has been investigated in the literature through the utilisation of several approaches to vagus stimulation, such as the Valsalva manoeuvre, stimulation of the vagus nerve from the ear region, and the application of invasive or non-invasive devices targeting the neck region [17,18]. 

Our research incorporates a novel approach to alleviate venepuncture discomfort by the application of cold stimulation to the vagus nerve. The findings of our investigation indicate that the administration of ice to the vagus nerve has a pain-reducing effect. In the course of our comprehensive literature review, we encountered a dearth of studies investigating the administration of analgesia via vagus nerve stimulation through the application of cold stimuli to the neck area.

This study has several limitations. Firstly, it was conducted in a single centre, which may restrict the generalisability of our findings in other settings. Additionally, the psychological status of the patients, including factors such as anxiety and depression, was not assessed prior to the trial. These psychological factors have been shown to potentially influence pain perception and might have impacted our results. The application of quantifiable markers for assessing nerve vagus stimulation could potentially enhance the effectiveness. An intriguing comparison could be made with a group receiving local anaesthesia.

In conclusion, in this study, we observed that patients whose necks underwent cold compress had a reduced pulse rate that remained low during vascular access. Furthermore, these patients experienced less discomfort during vascular access. The research presented in this study can be regarded as a valuable contribution to the existing body of knowledge on vagus stimulation techniques, which have been employed in several domains such as anxiety management, depression treatment, analgesic efficacy, and epilepsy therapy. To effectively transfer our findings into clinical practice, it is imperative to substantiate our research with studies conducted in diverse contexts such as critical care units, oncology services, and other relevant settings. Additionally, it is crucial to encompass a wide range of age groups, including children and the elderly, to ensure the applicability of our findings across various populations. Furthermore, it is essential to foster broad involvement in these studies to enhance the generalisability and robustness of our conclusions.

## Figures and Tables

**Table 1:** Demographic data.

	Group				*p*
		S (n = 52)	K (n = 54)	M (n = 56)	
Age (Mean ± SD)		47.25 ± 17.71	47.3 ± 15.84	44.54 ± 15.56	>0.05
Gender (n)	Male	24	21	24	>0.05
	Female	28	32	32	
BMI (Mean ± SD)		28.71 ± 5.7	28.97 ± 5.44	28.00 ± 5.27	>0.05
ASA (n) I/II/III		7/33/12	5/40/9	18/36/2	

BMI = body mass index; ASA = American Society of Anesthesiologists, SD = standard deviation, n = number.

**Table 2:** Changes in heart rates.

	Heart Rate Beats/min (Mean ± SD)		
	Initial Value	After Vagal Stimulation	*p*
M (n = 56)	77.07 ± 10.38	72.66 ± 10.29	<0.05
	**Initial Value**	**After Vascular Access**	
S (n = 52)	74.0 ± 12.56	76.98 ± 14.41	<0.05
K (n = 54)	76.46 ± 12.29	78.04 ± 12.14	>0.05
M (n = 56)	77.07 ± 10.38	76.02 ± 10.43	>0.05

n = number, SD: standard deviation.

**Table 3:** Evaluation of pain scores between groups.

	Groups			*p*
	S (n = 52)	K (n = 54)	M (n = 56)	
NRS (Mean ± SD)	5.02 ± 2.04	4.94 ± 1.72	^a^ 4.16 ± 1.97	<0.05
No Pain (NRS 0)	0	0	1	NA
Mild Pain (NRS 1–3)	14	10	^b^ 21	<0.05
Moderate/Severe Pain (NRS 4–10)	38	44	34	>0.05

NRS = numeric rating scale, n = number, SD = standard deviation, NA = Not applicable. ^a^ *p* < 0.05 when S-K-M, K-M, S-M groups were compared, ^b^ *p* < 0.05 when M and K groups were compared.

## Data Availability

The corresponding author can share data whenever desired.
